# Females Are More Anxious Than Males: a Metacognitive Perspective

**Published:** 2011

**Authors:** Fatemeh Bahrami, Naser Yousefi

**Affiliations:** 1 Department of Counseling, University of Isfahan, Isfahan, Iran; 2 Department of Psychology, University of Kurdistan, Kurdistan, Iran

**Keywords:** Anxiety Thoughts Inventory (AnTI), Generalized Anxiety Disorder (GAD), Metacognitive Thought Control (MTC), Worry

## Abstract

**Objective:** Previous studies have suggested that anxiety disorders are more prevalent among women than men. The purpose of this study was to compare the metacognitive perspective of Generalized Anxiety Disorder (GAD) in females with males.

**Methods:** A cross sectional study was conducted on the high school girls and boys who have been affected by GAD. The sample consisted of 100 high school students (50 boys and 50 girls) selected by Generalized Anxiety Disorder Scale (GADS) and semi-structured interview. They filled the Metacognitive Thought Control Questionnaire (TCQ), and Anxiety Thoughts Inventory (AnTI).

**Results:** Significant differences were observed between girls and boys in anxiety thoughts (health anxiety, social anxiety, and meta-worry). Anxiety thoughts affect girls more than boys; they have more metacognitive beliefs about uncontrollability of worry and believe that worry must be avoided. On the other hand, positive beliefs in worry are more common in girls and punishment and meta-worry are being implemented as control strategies in girls more than boys.

**Conclusion:** Metacognitive beliefs in uncontrollability, advantages and avoidance of worry may contribute to the higher prevalence of anxiety in females than males.

## Introduction

Generalized anxiety disorder (GAD), which can be viewed as the most normal anxiety disorder, is defined according to DSM-IV criteria (1) as an excessive anxiety and a subjectively uncontrollable worry in the presence of at least three somatic symptoms persisting for at least six months. The cognitive processes of GAD are similar to those in high trait anxiety (2). Therefore, understanding the underlying cognitive processes and mechanisms in this disorder can contribute to our general understanding of anxiety vulnerability.

GAD tends to be more frequent among women, blacks, young adults, and ones with low income or occupational status (3). The lifetime prevalence of GAD in the general population has estimated to be between 1.9% and 5.4%. In this regard, community surveys indicate a female to male preponderance of 2:1 in GAD. Moreover, multivariate logistic regression analysis indicated that being older than 24, having had a previous marriage (being separated, divorced, or widowed), and being a homemaker or unemployed are the significant correlates of the disorder (4,5).

A variety of researches have shown that anxiety disorders are more prevalent among women than men. However, these reports provoke the question: what can be influenced by using worry as a central factor of GAD and rumination as a central factor of depression in females? Hence, this study was performed to answer this question through metacognitive approach.

*A metacognitive model of GAD*

Wells has viewed GAD as one of the most basic manifestatic general maladaptive metacognitions that comprise vulnerability of psychological disorders (6,7). He has developed a model accounting for pathological worry in GAD. In this model, metacognition in the forms of belief appraisals and control strategies is the central factor to develop and maintain the disorder. The model differs from other cognitive conceptualizations of GAD by emphasizing the role of metacognition rather than maladaptive beliefs in world as a dangerous place. In this model, worry in GAD is not merely a symptomatic consequence of anxiety, but an active and motivated style of appraisal and coping with threat driven by the individual's beliefs. Indeed, worry is used in GAD in order to cope with anticipated dangers and threats. A distinction is made between two types of worrying, labeled type I and type II in this model. Type I worry is concerned with external and non-cognitive internal events (e.g. physical symptoms), whilst type II worry concerns negative appraisal of one's own thought processes. The positive metacognitive beliefs in the usefulness of worry as a coping strategy are activated and lead to activation of an inherent anxiety program and to cognitive and somatic symptoms consequently. A person with GAD continues to worry until the time that s/he will effectively be able to cope with an anticipated threat. This assessment is often based on internal cues such as a "felt sense" that one will be able to cope or the belief that all important outcomes have been considered in details. Worrying stops when these internal goals are met.

Worry type II problems (worry about worry or metaworry) are consequences of negative metacognitive beliefs in the worry process and the consequences of worrying. Individuals with GAD hold negative beliefs as well as positive ones about worrying (8). Experimental works support metacognition in emotional disorder. Positive and negative beliefs correlate positively with proneness to pathological worry (9,10).

Borkovec and Roemer (11) have shown that positive reasons of worrying including superstition and problem-solving are given higher ratings by individuals with GAD compared with non-anxious subjects. Greater negative beliefs in worrying are significantly reported in patients with GAD in comparison with patients with panic disorder and social phobia of non-patient controls. However, they show equivalent levels of positive beliefs (12). Type II worry is a better predictor of pathological worry in non-patients compared with type I (12). Besides, higher metaworry scores have been observed in GAD compared with panic disorder, social phobia, or non-patients (13). Worrying appears to be associated with intrusive thoughts under some circumstances (14). These data support the idea that using worry as a processing strategy may well contribute to the proliferation of intrusive thoughts under some circumstances.

The dimension of metacognition which is linked to psychological problems is used as a part of thought controls strategies. Studies of thought suppression, in which subjects attempt not to think about particular target thoughts, indicate that thought suppression of this kind can lead to an immediate and/or delayed increase in the target thought occurrence (15).

Some thought control strategies may be more effective than others and their effectiveness in or impacts on emotional well-being will be influenced by the context in which they are used and the purpose they serve. In particular, it has been suggested that some individuals, particularly those with GAD, use worry in order to distract from more upsetting images (16), as well as a means of coping with anticipated threats (6-7). Under specific conditions of short exposures to worry, worry tends to lead to an increase in intrusive thoughts as demonstrated experimentally (4, 17).

There are five different types of thought control strategies such as distraction e. g. "I do something that I enjoy.", social control e. g. "I ask my friends if they have similar thoughts.", worry e. g. "I focus on different negative thoughts.", punishment e. g. "I punish myself for thinking the thought" and reappraisal e. g. "I try to reinterpret the thought" (18). The tendency to use worry and punishment as control strategies is positively associated with measures of pathological worry, neuroticism and introversion. The other thought control subscales of distraction, social control and reappraisal show non-significant but negative correlations with stress vulnerability measure in the study of Wells and Davies (19).

*Gender and worry*

More evidences exist of individual differences in using rumination as a predictor of depression than individual worry. Investigating examines vulnerability to negative emotional states such as dysphoria, and GAD has increasingly got interested in how individuals respond to these moods. Individual differences in cognitive response to negative moods (such as dysphoria) are hypothesized to determine whether or not these moods persist and spiral into more severe and persistent clinical disorders (12).

People are different in the way they regulate their emotions; some seem to regularly engage in rumination and worry. To date, Nolen-Hoeksema and her colleagues (20) have garnered a strong support for her model across a variety of study designs with nonclinical samples. In an early test, they found that following a depressive mood induction, the individual who was assigned to a physically active distracting task exhibited the greatest alleviation of dysphoria mood. In contrast, the ones, assigned to a physically passive ruminative task, remained the most dysphoric. Similar results were observed in a natural nonclinical dysphoria (21).

It has been suggested that rumination might mediate the effects of other risk factors for dysphoria and depression. For example, the higher prevalence of depression in females than in males might be explained by the tendency of females to ruminate and worry.

However, rumination and worry do not seem to be adaptive emotion regulation strategies. The reason is that they persistently focus on negative cognitions and consequently, they appear to worsen one's depressive mood rather than alleviate it. Papageorgiou and Wells (22,23) offered an explanation by examining positive metacognitive beliefs in rumination in people with recurrent major depression. People holding positive metacognitive beliefs (such as believing in rumination as a helpful strategy for gaining insight, identifying causes and triggers of depression, solving problems, preventing future mistakes and failures and prioritizing important tasks) tended to ruminate more than individuals without such beliefs.

Moreover, rumination mediated the relation between the positive beliefs, state and trait depression in this sample. Lyubomirsky & Nolen Hoeksema (24) showed that ruminators believed that they were gaining greater psychological insight in response to dysphoria, whereas males tended to actively distract themselves from these negative moods. Therefore, the effect of gender is mediated, at least in part, by ruminative response styles (20). The researches that are conducted to explain gender differences in worry are limited.

This study hypothesized that females are more anxious because they believe that worry is useful and helps them to prevent future bad events and keep them aware of warning signs. When they become overwhelm in their worry, they believe this over-worrying is very dangerous and cannot be controlled and in this case, they suffer type II worry. In fact, men use distraction as a coping strategy more than women.

*What did this study explore?*

Our purpose was to determine the factors that cause anxiety disorders and are more prevalent among women than men.

## Materials and Methods

*A) Design:* A cross-sectional causal analysis was used in this study.

*B) Population and process of sampling:* This study included 600 male and female high-school-students of Isfahan in 2007-08. Simple sampling method was used to enroll the subjects. The advantage of this type of sample selection is that it increases the probability of sample being the referent of a bigger society. Four hundred subjects were randomly selected from high school students, 104 of whom have been diagnosed to suffer from GAD (53 girls and 52 boys) according to DSM-IV criteria and GADs. Our sample was given the questionnaires for which they provided answers in 60 minutes. In this phase, 4 of the questionnaires were answered incompletely and were omitted reducing the sample population to 100 (50 females and 50 males). Participants have the average age of 16.4 (ranging between 15 and 18 years).

*Research moral:* The subjects were told that these data are gathered for research purposes and they will be kept confidential. It was also announced that subjects can get further information on the results of this study via e-mail.

*D) Instruments:* The instruments used were as follows:

*1) AnTI *(25) *evaluates person's readiness toward worry and is provided by Wells* *(*25*).* This inventory evaluates anxiety thoughts in three scales: social, health and worry anxiety (metaworry).

*2) GADs* (7) that is used for metacognition extractions of GAD, assesses changes in behavioral, cognitive and emotional dimensions and includes positive metacognitive beliefs in worry, negative beliefs in uncontrollability of worry and avoidance of worry.

*3) Diagnostic standards based on DSM-IV for clinical interview and diagnosis of GAD patients* *(* 1 *)* *.* Clinical interview and diagnosis which were used in this study were developed by research authors’ based on DSM-IV.

*4) TCQ* is developed by Wells and Davies (19) to assess individual differences in the use of a range of thought control strategies. This scale is comprised of five subscales that measure thought control strategies of distraction, social control, worry, punishment and reappraisal.

These three questionnaires (AnTI, GADS, and TCQ) were administered to those subjects. They were asked to read the questions carefully and answer all of them. The questionnaires were given to the students, and were collected after some days. Then, the data were gathered for statistical analysis. Multivariate Analysis of Variance (MANOVA) was used to test the hypothesis.

## Results

The first hypothesis concerned with the differences between males and females in Health Anxiety, Metaworry, Social Anxiety, Worry Uncontrollability, Avoidance of Worry, Positive Beliefs about Worry, Punishment Strategy, Control Strategy, Social Control Strategy and Reappraisal Strategy.

The results presented in table 1 indicate that a significant difference was found (P=0.001) in the scores of variables between Males and Females.

Table 2 presents comparisons of anxiety thoughts based on gender. Gender is an independent variable and anxiety thought is a dependent one. Table 3 shows that thoughts related to health anxiety are significantly different between two genders (F= 13.76, P= 0.001) and the girls ( = 13.17) suffer from health anxiety more than the boys ( = 10.07).

There is a significant difference between the girls and the boys regarding anxiety thoughts related to metaworry (F=8.34, P=0.02) and the girls ( =15.27) are affected by anxiety thoughts related to metaworry more than the boys ( =11.30). It means that the girls have more positive and negative metacognitive beliefs about worry and this has increased pathological or type II worry.

**Table 1:** Multivariate analysis of variance (MANOVA), differences between the scores in males and females

Name of test	Value	F	df(H1,2,3)	df(fault)	P
Pillai's Trace	1.055	10.61	18	340	0.001
Wilks' Lambda	0.052	32.01	18	315	0.001
Hotelling's Trace	13.33	86.23	18	318	0.001
Roy's Largest Root	14.12	268.10	6	111	0.001

**Table 2:** Comparison of health anxiety thoughts between males and females

Source of Variation	Ss	df	MS	F	P
Between Groups	162	1	162	13.76	0.001
Within Groups	953	58	16.32		
Total	1115	59			

**Table 3:** Comparison of metaworry thoughts between males and females

Source of Variation	Ss	df	MS	F	P
Between Groups	248.13	1	248.13	8.34	0.02
Within Groups	2819.34	58	23.43		
Total	3067.47	59			

Table 4 shows that there is a significant difference between the girls’ and the boys’ social anxiety thoughts (P< 0.01, F=11.68). Furthermore, the girls suffer from social anxiety ( =22.5) more than the boys ( =18.17). The second hypothesis concerned with the differences between males and females in metacognitive beliefs.

**Table 4:** Comparison of social anxiety thoughts between males and females

Source of Variation	Ss	df	MS	F	P
Between Groups	395.3	1	395.3	11.68	0.001
Within Groups	102.3	58	47.6		
Total	4917.6	59			

Table 5 presents the variance analysis of metacognitive beliefs according to gender. According to the table 4, there was a significant difference between the girls and the boys in metacognitive beliefs about avoidance of worry (F=6.56, P= 0.002), and the girls ( =50.4) had negative metacognitive about avoidance more than the boys ( =30.5).

**Table 5:** Variance analysis of metacognitive beliefs about uncontrollable worry in males and females

Source of Variation	Ss	df	MS	F	P
Between Groups	22.88	1	22.88	6.56	0.002
Within Groups	235.8	58	40.6		
Total	4917.6	59			

Due to the table 6, it can be concluded that there was a significant difference between the girls and the boys in negative metacognitive beliefs about uncontrollable worry (F=9.34, P=0.02). The girls ( =5.2) more than the boys ( =3.9) believed that worry is uncontrollable.

**Table 6:** Variance analysis of metacognitive beliefs related to avoidance of worry in males and females

Source of Variation	Ss	df	MS	F	P
Between Groups	653.02	1	653.02	9.34	0.002
Within Groups	5116.61	58	660		
Total	5769.62	59	69.401		

Table 7 shows that there was a significant difference between the boys and the girls about positive beliefs related to worry (F=21.44, P=0.01). The girls ( =50.4) more than the boys ( =30.5) believed that worry could be useful and their metacognitive beliefs about worry were positive.

**Table 7:** Variance analysis of metacognitive beliefs related To positive beliefs about worry in males and females

Source of Variation	Ss	df	MS	F	P
Between Groups	5421.3	1	5421.3	21.44	0.01
Within Groups	13314.1	58	264.5		
Total	18735.4	59			

Table 8 presents the variance analysis of thought control strategies and shows a significant higher rate of using punishment strategy in the girls than the boys (P<0.01, F=14.7).

Table 9 shows a higher rate of using worry strategy in the girls than the boys (P<0.00, F= 19.45).

Table 10 shows that there were no significant differences between the girls and the boys in using distraction strategy (P=0.03, F= 1.89).

**Table 8:** Variance analysis of punishment strategy in males and females

Source of Variation	Ss	Df	MS	F	P
Between Groups	111.2	1	111.2	14.7	0.001
Within Groups	743.3	8	9.1		
Total	854.5	59			

**Table 9:** Variance analysis of control strategy in males and females

Source of Variation	Ss	Df	MS	F	P
Between Groups	333.2	1	333.2	19.45	0.000
Within Groups	854.4	58	12.3		
Total	1187.6	59			

**Table 10:** Variance analysis of control strategy in males and females

Source of Variation	Ss	Df	MS	F	P
Between Groups	3.51	1	3.51	1.89	0.003
Within Groups	631.21	58	7.86		
Total	634.72	59			

Nonetheless, no significant difference was observed between the girls and the boys regarding using social control strategy(P=0.001, F=2.32) (Table 11).

**Table 11:** Variance analysis of control strategy in males and females

Source of Variation	Ss	Df	MS	F	P
Between Groups	7.43	1	7.43	2.32	0.001
Within Groups	532.16	58	6.48		
Total	539.59	59			

Table 12 expresses no significant differences between the girls and the boys in using reappraisal strategy (P=0.002, F= 1.12).

**Table 12:** Variance analysis of reappraisal strategy in males and females

Source of Variation	Ss	df	MS	F	P
Between Groups	7.21	1	7.21	1.12	0.002
Within Groups	537.36	58	6.45		
Total	544.57	59			

Based on our results, there was a significant relation between the girls' health anxiety thoughts and uncontrollability of worry (Rxy=0.45, P<0.01), and between social anxiety thoughts and metaworry (Rxy=0.38, P<0.05). It means that a person who believes in uncontrollability of worry suffers from social anxiety, health anxiety and metaworry (worry about worry) more than others.

## Discussion

The results have shown a higher rate of health anxiety and metaworry in girls than boys. Besides, it can be inferred that girls more than boys believe that worry is uncontrollable and must be avoided due to metacognition. There is a relation between health anxiety and metacognitive beliefs about uncontrollability of worry. It means that if an individual thinks that worry is uncontrollable, s/he is affected by health and social anxiety more considerably. Girls have metaworry more than boys because metacognitive beliefs about uncontrollability of worry are more prevalent in girls. It means that girls believe that worry is uncontrollable. As a result, they worry about their worry and suffer from type II worry. There is a relation between avoidance of worry and metaworry in boys and girls. It means that if they believe that worry is dangerous and must be avoided, they are affected by type II worry.

The results of this research are consistent with the results of Wells and Carter's study (13). They presented a significant relation between social worry, health worry and metaworry and generalized anxiety. Furthermore, Wells and Papageorgiou (26,27), Cartwright-Hatton, Wells (9), Wells & Papageorgiou (24), and Borkovec & Romer (11) found that positive and negative beliefs correlate positively with proneness to pathological worry. Borkovec, Robinson, Pruzinsky & Depree (14), York, Borkovec, Vasey, & Stern (28), Butler, Wells, & Dewick (17) support this idea that worrying appears to be associated with increased intrusive thoughts under some circumstances.

Wells and Carter (12) examined type II worry and metacognitive beliefs in patients with GAD, social phobia, panic disorder, and individual with no history of disorder. Patient with GAD differed from other anxious groups in reporting higher levels of metaworry and negative beliefs about worrying. There were no differences between those groups in positive beliefs. Patients with depression showed some metacognitive similarity to GAD patients. These data are consistent with a central prediction of GAD patients that they should be characterized by metaworry and negative beliefs.

The reason for this particular sequence is that when patients believe that worrying is uncontrollable, it is often too threatening for them to comply optimally with behavioral experiments consisting of attempts to "lose control" of the worry process. Negative metacognitions should be targeted in therapy before positive beliefs, since these are most closely linked to acute anxiety.

Moreover, the results suggest that girls use punishment (P=0.001) and metaworry (P=0.00) of thought control strategies more than boys, but there are not any significant differences between girls and boys in distraction (P=0.07), social control(P=0.28) and reappraisal (p=0.33). Wells and Davies (19) have suggested that the use of worry and punishment to control unwanted thoughts is associated with proneness to emotional problems. It is possible that other thought control strategies, i.e. social control, reappraisal and distraction which appear not to correlate with neuroticism or trait anxiety significantly may be positive psychological health markers that buffer against emotional vulnerability under some circumstances (8,29).

## Conclusions

The results showed that girls are more prone to anxiety than boys because of their though control strategies and metacognitive beliefs, which lead them to emotional and neurotic problems. Therefore, practice of alternative strategies for threat processing should be taught to them. According to this study, we can believe that girls need to learn more about the ways that help them to control their metacognitive worrying thoughts and modify their negative and positive metacognitive beliefs about worry.

## Authors' Contributions

FB conceived and designed the evaluation and helped to draft the manuscript, collected the clinical data, interpreted them and revised the manuscript. NY participated in designing the evaluation and performed parts of the statistical analysis, revised the manuscript and performed the statistical analysis and revised the manuscript, interpreted them. Both authors read and approved the final manuscript.

## Notes

**Declaration of Interest:** None.

Iran J Psychiatry Behav Sci 2011; 5(2): 83-90.

## References

[1] American psychiatric Association (APA) (1994). Diagnostic and Statistical Manual of Mental Disorder, Revised.

[2] Eysenck MW (1997). Anxiety and cognition: A unified theory.

[3] Lindemann C (1996). Handbook of the treatment of the anxiety disorders.

[4] Wittchen HU, Zhaos S, Kessler RC, Eaton WW (1994). DSM III-R generalized anxiety disorder in the National Comorbidity Survey. Arch Gen Psychiatry.

[5] Blazer D, George LK, Hughes D, Salzman C, Lebowitz BD (1991). The epidemiology of anxiety disorders: an age comparison. Anxiety in the elderly: Treatment and research.

[6] Wells A (1995). Meta-cognition and worry: a cognitive model of generalized anxiety disorder. Behav Cogn Psychoter.

[7] Wells A (1997). Cognitive Therapy of Anxiety Disorders: A practice manual and conceptual Guide.

[8] Wells A (2000). Emotional disorders metacognition (innovative cognitive therapy).

[9] Cartwright HS, Wells A (1997). Beliefs about worry and intrusions: the metacognitions Questionnaire. J Anxiety Disord.

[10] Wells A, Papageorgiou C (1998). Relationships between worry and obsessive- compulsive symptoms and metacognitive beliefs. Behav Res Ther.

[11] Borkovec TD, Roemer L (1995). Perceived function of worry among generalized anxiety subjects: distraction from more emotionally distressing topics?. J Behav Ther Exp Psychiatry.

[12] Wells A, Carter K (2001). Further tests of a cognitive model of generalized anxiety disorder: worry and metacognitions in patients with generalized anxiety disorder, Panic disorders, Social phobia, and depression. Behav Ther.

[13] Wells A, Carter K (1999). Preliminary tests of a cognitive model of generalized anxiety disorder. Behav Res Ther.

[14] BorkovecvT D, Robinson E, Pruzinsky T, Depree JA (1983). Preliminary exploration of worry: some characteristics and processes. Behav Res Ther.

[15] Wegner DM, Shortt JW, Blake AW, Page MS (1990). The suppression of exciting thoughts. J Pers Soc Psychol.

[16] Brokovec TD, Inz J (1990). The nature of worry in Generalalized Anxiety disorder: a predominance of thought activity. Behav Res Ther.

[17] Butler G, Wells A, Dewick H (1995). Differential effects of worry and imagery after exposure to a stressful stimulus: a pilot study. Behav Cogn Psychother.

[18] Teasdale JD (1983). Negative thinking in depression: cause, effect, or reciprocal relationship?. Adv Behav Res Ther.

[19] Wells A, Davies M (1994). The Thought Control Questionnaire: a measure of individual differences in the control of unwanted thoughts. Behav Res Ther.

[20] Nolen HS, Morrow J (1993). Effects of rumination and distraction on naturally occurring depressed mood. Cogn Emot.

[21] Morrow J, Nolen-Hoeksema S (1990). Effects of responses to depression on the remediation of depressive affect. J Pers Soc Psychol.

[22] Papageorgiou C, Wells A (2001). Metacognitive beliefs about rumination in recurrent major depressionm. Cogn Behav Pract.

[23] Papageorgiou C, Wells A (2001). Positive beliefs about depressive rumination: Development and preliminary validation of a self-report scale. Behav Ther.

[24] Lyubomirsky S, Nolen- Hoeksema S (1993). Self-perpetuating properties of dysphoria rumination. J Pers Soc Psychol.

[25] Wells A (1994). A multidimensional measure of worry: development and preliminary validation of the anxious thoughts Inventory. Anxiety Stress Coping.

[26] Wells A, Papageorgiou C (1998). Social phobia: effects of external attention on anxiety, negative beliefs, and perspective thinking. Behavior Therapy.

[27] Wells A, Papageorgiou C (1995). Worry and the incubation of intrusive images following stress. Behav Res Ther.

[28] York D, Borkovec TD, Vasey M, Stern R (1987). Effects of worry and somatic anxiety induction on thoughts, emotion and physiological activity. Behav Res Ther.

[29] Wells A (1999). A metacognitive model and therapy for generalized anxiety disorder. Clin Psychol Psychother.

